# Quantitative Bioluminescent Imaging of Pre-Erythrocytic Malaria Parasite Infection Using Luciferase-Expressing *Plasmodium yoelii*

**DOI:** 10.1371/journal.pone.0060820

**Published:** 2013-04-11

**Authors:** Jessica L. Miller, Sara Murray, Ashley M. Vaughan, Anke Harupa, Brandon Sack, Michael Baldwin, Ian N. Crispe, Stefan H. I. Kappe

**Affiliations:** 1 Seattle Biomedical Research Institute, Seattle, Washington, United States of America; 2 Department of Global Health, University of Washington, Seattle, Washington, United States of America; 3 Institute of Biology, Freie Universitaet Berlin, Berlin, Germany; 4 Department of Pathology, University of Washington, Seattle, Washington, United States of America; Université Pierre et Marie Curie, France

## Abstract

The liver stages of *Plasmodium* parasites are important targets for the development of anti-malarial vaccine candidates and chemoprophylaxis approaches that aim to prevent clinical infection. Analyzing the impact of interventions on liver stages in the murine malaria model system *Plasmodium yoelii* has been cumbersome and requires terminal procedures. *In vivo* imaging of bioluminescent parasites has previously been shown to be an effective and non-invasive alternative to monitoring liver stage burden in the *Plasmodium berghei* model. Here we report the generation and characterization of a transgenic *P. yoelii* parasite expressing the reporter protein luciferase throughout the parasite life cycle. *In vivo* bioluminescent imaging of these parasites allows for quantitative analysis of *P. yoelii* liver stage burden and parasite development, which is comparable to quantitative RT-PCR analysis of liver infection. Using this system, we show that both BALB/cJ and C57BL/6 mice show comparable susceptibility to *P. yoelii* infection with sporozoites and that bioluminescent imaging can be used to monitor protective efficacy of attenuated parasite immunizations. Thus, this rapid, simple and noninvasive method for monitoring *P. yoelii* infection in the liver provides an efficient system to screen and evaluate the effects of anti-malarial interventions *in vivo* and in real-time.

## Introduction

Malaria, caused by infection with *Plasmodium* species, continues to be a major burden to human health as it is responsible for 300–500 million infections and the deaths of 800,000 people annually [1]. There is currently no effective vaccine to prevent malaria, and new anti-malarial drugs are urgently needed to combat the increasing number of drug resistant *Plasmodium* strains. *Plasmodium* infection is initiated following the bite of an infected mosquito, when sporozoites are inoculated into the dermis. These sporozoites actively migrate to the blood stream to allow for passive transport to the liver, where they cross the sinusoidal endothelium and numerous hepatocytes until finally invading a terminal hepatocyte within which they grow and develop as liver stages (Reviewed in [2]). Each parasite undergoes replication in a host hepatocyte, leading to an enormous increase in parasite biomass and culminating in the release of 10,000–50,000 infectious exoerythrocytic merozoites into the blood. These merozoites initiate the asexual erythrocyte replication cycle responsible for the pathological manifestations of malaria [2]. In contrast to blood stage infection, when parasite numbers are very large, relatively few parasites productively establish infection in hepatocytes under natural transmission conditions. Thus, the liver stage of development is considered a bottleneck in the parasite’s lifecycle and has been an attractive target for vaccine development. Furthermore, genetically or radiation attenuated parasites that arrest during liver stage development are effective experimental vaccines, conferring complete sterile protection in rodent models of malaria [3], establishing a gold standard for protection and allowing analysis of protective immune mechanisms.

Quantitative analysis of liver stage burden is necessary for the study of drug and vaccination interventions that target pre-erythrocytic parasites. Standard methods of monitoring liver stage burden include quantitative RT-PCR (qPCR) to detect parasite ribosomal RNA, microscopy-based detection utilizing fluorescent parasites or immunofluorescence assays (IFA). However, these assays require liver extraction and are thus terminal. Therefore these assays have been confined to *in vitro* or *ex vivo* analyses. Bioluminescent assays have long been utilized to detect and monitor pathogens *in vitro* using plate-based luminometers. The development of *in vivo* imaging systems, which can detect, image, and quantify luminescent and fluorescent signals from anesthetized animals, now allows for the imaging of bioluminescent *Plasmodium* parasites *in vivo*. Indeed, infections of the mouse liver with bioluminescent strains of *P. berghei* and *P. yoelii* YM (a lethal strain) have previously been measured using bioluminescent imaging [4]–[7], and has been useful in monitoring the efficacy of drugs and immunizations. However, the currently available bioluminescent rodent malaria strains have a limited use because their bioluminescent signal cannot be detected at early time points after infection. This is especially true for the luciferase expressing *P. yoelii* YM strain, which was not detectable in the liver until 42 hours following infection with sporozoites and not detectable at all at low doses of infection (<1,000 sporozoites) [4]. Therefore, these parasites are sub-optimal for both monitoring parasite growth over time, and measuring low-dose sporozoite challenges. Finally, many malaria vaccine models, such as genetically attenuated parasites (GAPs), have been developed using the non-lethal *P. yoelii* XNL strain rather than the lethal YM strain [8]–[10] and thus an *P. yoelii* XNL-luciferase strain will be valuable to evaluate protective vaccination.

We have generated and characterized a transgenic *P. yoelii* XNL parasite that constitutively expresses a GFP-luciferase fusion protein (Py-GFP-luc) throughout the life cycle. While a GFP expressing bioluminescent *P. yoelii* XNL transgenic parasite was recently developed, this parasite has not been analyzed during liver or mosquito stages of development [11]. Using bioluminescent imaging, both liver stage burden and development of Py-GFP-luc could be monitored from as early as 16 hours post infection (hpi), and used to compare infectivity in different mouse strains. Finally, we show that this method is valuable in assessing the efficacy of active immunization regimens that target the pre-erythrocytic stages of infection.

## Results

### Generation of Py-GFP-luc

**Figure 1:**
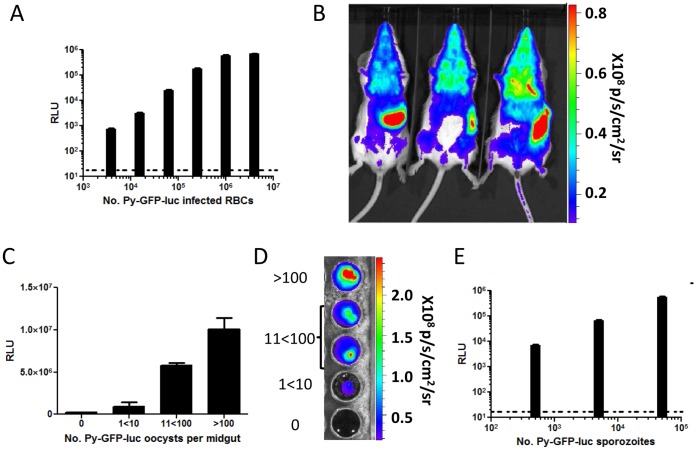
Luciferase activity of the Py-GFP-luc parasite. (A, B) Luminescence during erythrocytic stages. (A) Py-GFP-luc infected red blood cells were serially diluted 1∶4 in RPMI and were mixed with an equal volume of luciferin. Luminescence (relative light units, RLU) was measured with a Centro microplate reader. Dashed line indicates background luminescence as determined by measuring uninfected RBCs. (B) Rainbow images of luminescence in BALB/cJ mice with Py-GFP-luc blood stage parasitemia. Mice were injected with luciferin and imaged. Bioluminescence was measured in total flux (p/s). (C, D) Luminescence of Py-GFP-luc oocysts. (C) Midguts from Py-GFP-luc infected mosquitoes were isolated 10 days after an infectious blood meal. The number of oocysts per midgut was estimated and midguts were categorized as follows: Uninfected (0), between 1 and 10 oocysts/midgut (1<10), between 11 and 100 oocysts/midgut (11<100), and greater than 100 oocysts/midgut (>100). Luciferase activity from individual midguts was determined using the Centro microplate reader (RLU) (C) and the IVIS animal imager (total flux) (D). (E) Luminescence of Py-GFP-luc sporozoites. Sporozoites were isolated from salivary glands of infected mosquitoes and serially diluted 1∶10 in RPMI. Luminescence (RLU) was determined as in (A). Dashed line indicates background luminescence of RPMI.

We generated a transgenic *P. yoelii* XNL parasite expressing a GFP-luciferase fusion protein (Py-GFP-luc). The GFP-luciferase fusion construct, under the control of the constitutive promoter EF1α from *P. berghei*, was introduced into the dispensable S1 locus [12] by double homologous recombination (Figure S1A). Genotyping of four clones by PCR revealed double crossover homologous recombination of the construct and the absence of the S1 open reading frame (Figure S1B), demonstrating successful integration of the construct. Integration of the GFP-luc construct did not affect midgut or salivary gland sporozoite production or liver stage development as determined by time to blood stage patency (Table S1). Py-GFP-luc also showed normal blood stage replication (Figure S2). Luciferase was expressed throughout the blood and mosquito life cycle stages of the parasite (Figure 1). Luciferase activity was detected in a linear, dose-dependent fashion in mixed blood stages *ex vivo* using a plate-based luminometer assay (Figure 1A). Systemic blood stage infection was also readily observed by imaging bioluminescence in live, Py-GFP-luc infected Swiss Webster (SW) mice (Figure 1B). Bioluminescent signal in Py-GFP-luc blood stage-patent mice was most intense in the region of the spleen and the lungs (Figure 1B), which could be due to the sequestration of parasites in these organs [13]. Luciferase expression was also monitored in midgut oocysts (Figure 1C, D) and salivary gland sporozoites (Figure 1E). In a plate-based luminescence assays (Figure 1C, E), relative light units (RLU) corresponded to the number of Py-GFP-luc parasites, as RLUs increased with the concentration of parasites. Bioluminescence of mosquito midguts containing different numbers of Py-GFP-luc oocysts was also observed *ex-vivo* in a whole animal bioluminescence imager (Figure 1D).

### Analysis of Py-GFP-luc Liver-stage Burden *in vivo*

**Figure 2:**
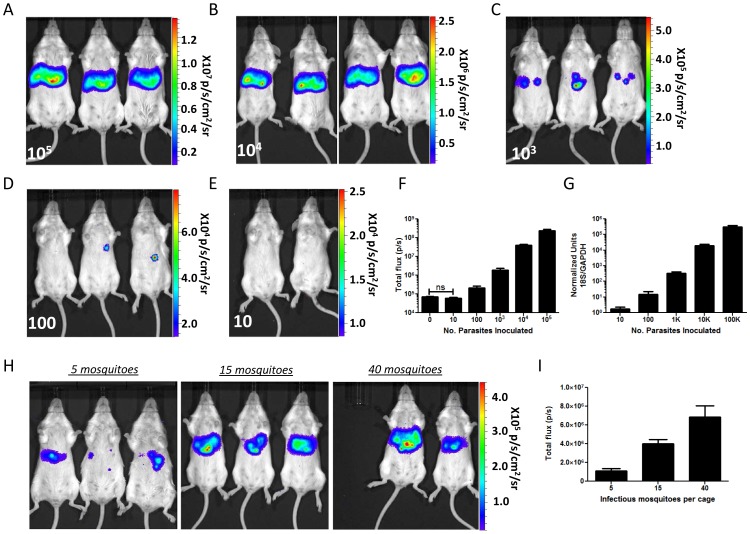
Luminescence is proportional to Py-GFP-luc liver stage burden *in vivo.* (A–E) Representative rainbow images of luminescence in livers of live mice 44 h following injection with (A) 1×10^5^, (B) 1×10^4^, (C) 1×10^3^, (D) 100 or (E) 10 Py-GFP-luc sporozoites. Rainbow scales are expressed in radiance (p/s/cm^2^/sr). (F) Quantification of total flux from mice in (A–E) (n = 5). (G) Quantification of Py-GFP-luc liver stage burden from dissected livers of mice in (A–E) by qPCR. Ratios of *P. yoelii* 18S rRNA to murine GAPDH RNA were calculated and normalized to that of uninfected mice. (H-I) Liver stage burden following mosquito bite infection. (H) Representative rainbow images of luminescence in livers of BALB/cJ mice 44 h after being fed on by 5, 15, or 40 mosquitoes infected with Py-GFP-luc. Rainbow scales are expressed in radiance (p/s/cm^2^/sr). (I) Quantification of total flux from mice in (H). n = 5 mice per group.

In order to evaluate pre-erythrocytic infection *in vivo*, groups of BALB/cJ mice (n = 4–6) were infected intravenously (i.v.) with increasing numbers of Py-GFP-luc sporozoites ranging from 10 to 10^5^. Forty-four hours post infection (hpi), when liver stage parasites have almost completed development and have reached near-maximum biomass, mice were injected with luciferin and liver stage luciferase activity was visualized and measured (Figure 2A–E). Bioluminescence was detected from all of the mice infected with the highest dose (1×10^5^ sporozoites) in the region of the liver, with a total flux (expressed as photons (p)/second (s)) of 2.4×10^8^ (Figure 2A, F). A ten-fold decrease in inoculum yielded a comparable decrease in luminescence, as infection with 10^4^, 10^3^, and 100 sporozoites resulted in a total flux of 4.1×10^7^, 2.0×10^6^, and 2.2×10^5^, respectively (Figure 2B–D, F). These data indicate that the flux is proportional to liver stage burden. None of the mice infected with the lowest dose of sporozoites (10) produced a signal over background in this experiment (Figure 2E, F). Correspondingly, a subset of these mice (n = 3) did not develop a blood stage infection when monitored for 14 days post infection (data not shown). Livers from a subset of mice were removed and their RNA extracted, reverse transcribed and subjected to qPCR using parasite 18S ribosomal RNA for the determination of parasite burden (Figure 2G). The relative luminescence intensities of mice infected with different sporozoite numbers was comparable to the relative amount of parasite 18S ribosomal RNA as determined by qPCR (Pearson’s correlation, r = 0.87, P = 0.01). We also assayed liver stage burden of Py-GFP-luc following the bites of infected mosquitoes, the natural route of infection. BALBc/J mice were exposed to 5, 15, or 40 infectious mosquito bites (average of approximately 32,000 sporozoites/mosquito salivary gland), which were allowed to feed for 10 minutes. Liver stage burden was determined 44 hpi by bioluminescent imaging (Figure 2H and I). Bioluminescence was detected in all of the mice, with a total flux of 1.1×10^6^, 4.0×10^6^ and 6.9×10^6^ for mice infected by 5, 15, and 40 mosquito bites, respectively (Figure 2H).

### Developmental Progression of Py-GFP-luc Liver Stages can be Monitored *in vivo* Using Bioluminescent Imaging

**Figure 3:**
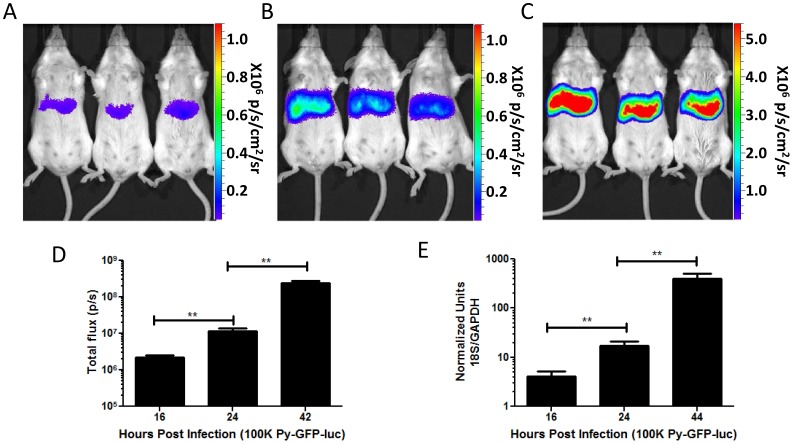
Luminescence correlates to Py-GFP-luc liver stage growth *in vivo*. (A–C) Representative rainbow images of luminescence in livers of live mice injected i.v. with 1×10^5^ Py-GFP-luc salivary gland sporozoites at (A) 16, (B) 24 and (C) 44 hpi. Rainbow scales are expressed in radiance (p/s/cm^2^/sr). (D) Quantification of total flux from mice in (A–C) (n = 5). (E) Quantification of parasite burden in the livers by qPCR of mice injected with 1×10^5^ Py-GFP-luc salivary gland sporozoites at various time points post infection (n = 3). Ratios of *P. yoelii* 18S rRNA to murine GAPDH RNA were calculated and normalized to that of uninfected mice. Statistics (B, C) Student’s *t* test, **indicates a P value of 0.01>0.001.

*P. yoelii* undergoes significant growth over the course of liver stage development and thus, due to the fact that luciferase expression is driven by the EF1α promoter, the amount of luciferase produced by Py-GFP-luc liver stages should increase over time. In order to determine if bioluminescence of Py-GFP-luc could be used to monitor liver stage growth, mice were infected i.v. with 1×10^5^ Py-GFP-luc sporozoites and bioluminescence in the liver was monitored at 16, 24, and 44 hpi (Figure 3). Luciferase activity was readily detectable at 16 hpi with a total flux of 2.23×10^5^ (Figure 3A, D). Total flux increased at 24 hpi to 1.14×10^7^ (Figure 3B, D) and to 2.4×10^8^ by 44 hpi (Figure 3C, D). As before, the increase of bioluminescent signal was comparable to the increase of 18S ribosomal RNA over time as determined by qPCR (Figure 3E) (Pearson’s correlation, r = 0.99, P = 0.0001).

### Analyzing Susceptibility of Different Mouse Strains to Py-GFP-luc Using Bioluminescent Imaging and qPCR

**Figure 4:**
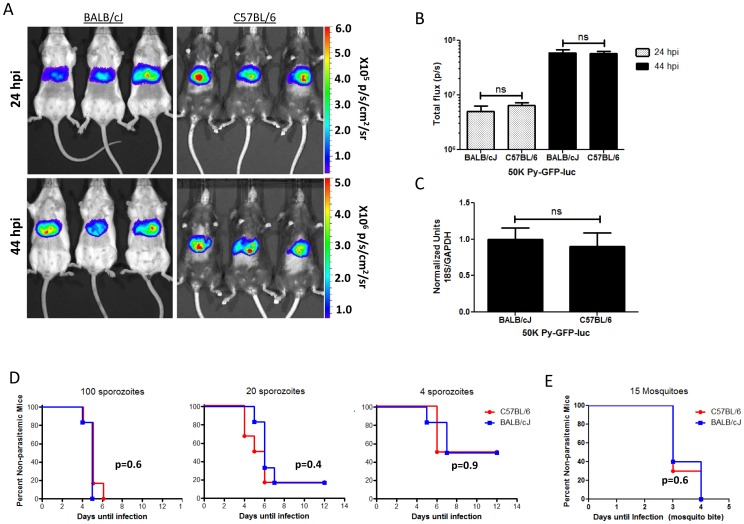
Use of bioluminescence to compare Py-GFP-luc liver stage burden in two mouse strains. (A) BALB/cJ or C57BL/6 mice (n = 4) were infected with 5×10^4^ Py-GFP-luc sporozoites i.v. The bellies of all the mice were shaved before imaging bioluminescence. Representative rainbow images are shown. Scale bar is in radiance (p/s/cm^2^/sr). (B) Quantification of parasite liver burden as determined by bioluminescence. (C) Quantification of parasite liver burden as determined by qPCR. Ratios of *P. yoelii* 18S rRNA to murine GAPDH RNA were calculated and normalized to that of BALB/cJ mice. (D, E) Days to patency comparisons between BALB/cJ and C57BL/6 mice. (D) BALB/cJ or C57BL/6 mice (n = 10) were infected i.v. with 100 (left), 20 (center) or 4 (right) *P. yoelii* sporozoites and blood stage patency was monitored in mice by daily thin smears. Graphs depict the percentage of mice free of blood stage malaria at each day post infection. (E) BALB/cJ or C57BL/6 mice were infected via the bites of either 15 (left) or 5 (right*) P. yoelii* infected mosquitoes. Blood stage patency was monitored and depicted as in (D). Statistics (B, C) Student’s *t* test ns = p>0.05. (D, E) Log rank Mantel-Cox test. P values are depicted in the figure.

Rodent malaria models have traditionally paired C57BL/6 mice with *P*. *berghei* infection and BALB/cJ mice with *P. yoelii* infection. To determine if these different mouse strains have different susceptibilities to infection with Py-GFP-luc, we infected both C57BL/6 and BALB/cJ mice with 5×10^4^ Py-GFP-luc sporozoites and measured liver stage burden at 44 hpi by bioluminescent imaging (Figure 4A). Since C57BL/6 mice have highly pigmented hair that absorbs the luciferase signal (data not shown), the mice were shaved prior to imaging. Liver stage burdens in C57BL/6 and BALB/cJ mice were not statistically different from each other as measured by luciferase activity (Figure 4B) and by qPCR (Figure 4C).

While liver stage burdens of Py-GFP-luc parasites were not significantly different between BALB/cJ and C57BL/6 mice following infection with 5×10^4^ sporozoites, it is possible that differences in susceptibilities of these strains may only be observed at low sporozoite doses. Thus, we compared the ability of low doses of infectious sporozoites to establish blood stage patency in these strains. Mice were infected i.v. with 4, 20, or 100 salivary gland *P. yoelii* sporozoites and blood stage parasitemia was determined daily by Giemsa-stained thin blood smears starting at 3 days post infection (Figure 4D). An inoculum of 100 sporozoites consistently induced blood stage patency in both strains starting at 4 days pi with the median day to patency being 5 days pi. A dose of 20 sporozoites induced a blood stage infection in 80% of BALB/cJ and C57BL/6 mice, with the median day to patency being 5 and 6, respectively. The lowest infectious dose of 4 sporozoites resulted in blood stage infection in only 50% of mice from both groups between day 6 and 8 post infection (Figure 4D). Rate and frequency of infection between the two strains of mice was not significantly different for any of the three infectious doses (log rank Mantel-Cox test, 100: P = 0.60; 20: P = 0.43; 4: P = 0.92). To determine if these mouse strains are also equally susceptible to *P. yoelii* infection via the natural route of mosquito bite, fifteen infected mosquitoes (average of approximately 44,000 sporozoites/mosquito) were allowed to feed on BALB/cJ or C57BL/6 mice for 10 minutes. All the mice developed blood stage infection (median day to patency = 3) (log rank Mantel-Cox test, P = 0.65) (Figure 4E).

### Evaluating Efficacy of Vaccination Using Bioluminescent Imaging

**Figure 5:**
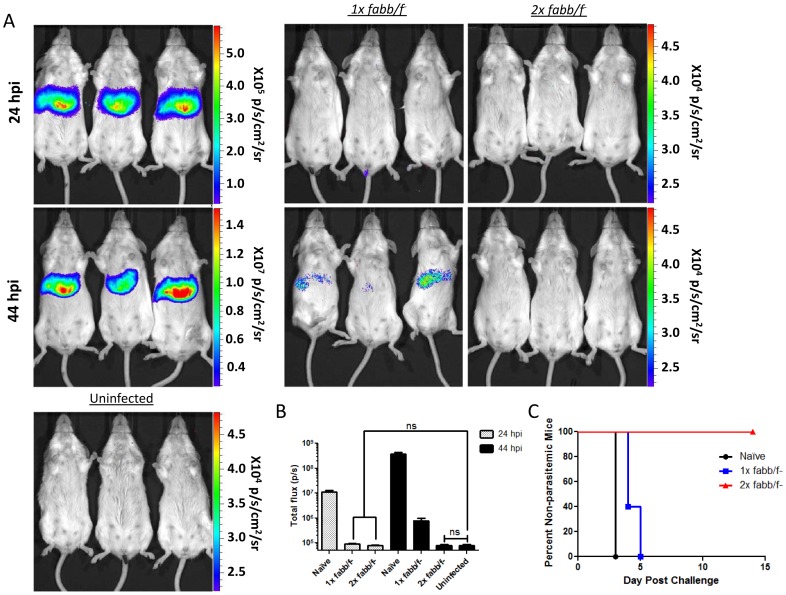
Use of bioluminescence to evaluate the efficacy of GAP immunization. BALB/cJ mice were immunized with either 1 or 2 doses of the GAP *Py-fabb/f-*. Immunizations were spaced 3 weeks apart in multiply immunized mice. Four weeks after the final immunization naïve and immunized mice were challenged with 5×10^4^ Py-GFP-luc sporozoites by i.v. injection. Bioluminescence was measured 24 hpi and 44 hpi. Mice were then monitored for blood stage patency. (A) Representative rainbow images of naïve, or immunized mice challenged with Py-GFP-luc (n = 6). Uninfected mice were also imaged as a control (n = 5). Scale bars are in radiance (p/s/cm^2^/sr). (B) Quantification of parasite liver burden by bioluminescence. (C) Blood stage patency in challenged mice was monitored from days 3 to 14 post infection by daily thin smears. Graph depicts the percentage of mice free of blood stage malaria at each day post infection.

Bioluminescent imaging has previously been utilized to monitor the efficacy of immunization with chemical and radiation attenuated *P. berghei* sporozoites [5]. However, this method was not used to monitor the efficacy of immunization with genetically attenuated parasites (GAPs). FabB/F deficient GAPs (*Py-fabb/f-*), which carry a gene deletion in the apicoplast-targeted fatty acid biosynthesis pathway, arrest late during liver stage development and persist in the liver for up to 60 hpi [14]. Immunization of mice with *Py-fabb/f-* completely protects mice from sporozoite challenge [8]. To determine if bioluminescent imaging could be utilized to monitor the efficacy of GAP vaccination, BALB/cJ mice (n = 5) were immunized once or twice with 5×10^4^ *Py-fabb/f-* sporozoites. Prime-boost immunizations were three weeks apart and all mice were challenged with 5×10^4^ Py-GFP-luc sporozoites four weeks after the final immunization. Protection was evaluated via bioluminescent imaging at 24 hpi and 44 hpi (Figure 5A, B) and the reading of blood smears on days 3–14 post infection (Figure 5C). Five naïve control mice were also infected with Py-GFP-luc at the time of challenge. All of the control challenged mice became patent for blood stage parasites 3 days post challenge and a robust bioluminescent signal was observed in the livers at 24 hpi and 46 hpi (Figure 5A and B). All of the mice immunized twice with *Py-fabb/f-* were protected from malaria, as they never developed blood stage infection (Figure 5C). Furthermore, the bioluminescent signal detected in the livers of these mice was not significantly different from uninfected mice (8.4×10^4^ and 7.9×10^4^ p/s, respectively) (Figure 5A, B). Mice immunized with only a single dose of *Py-fabb/f-* were partially protected from a challenge with 5×10^4^ Py-GFP-luc sporozoites, as reduced bioluminescent signal (7.8×10^5^ p/s) was detected in the liver at 46 hpi and all mice became patent by five days pi, a 2-day delay in patency (Figure 5A–C).

## Discussion

The pre-erythrocytic stages of malaria parasite development are promising targets for anti-malarial vaccination and prophylaxis as they represent a major bottleneck in the parasite life cycle. The rodent infective *P. yoelii* XNL parasite is an important model organism for malaria, and has been used in multiple studies to identify and evaluate anti-malarial vaccines and therapeutics. However, until recently it was not possible to analyze *P. yoelii* XNL liver stage infection noninvasively and in real-time. Here, we have developed a transgenic *P. yoelii* XNL parasite expressing a GFP-luciferase fusion protein that can be used to noninvasively monitor *P. yoelii* liver stage infection using whole-body bioluminescent imaging. GFP-luciferase is expressed constitutively throughout the parasite’s life cycle under the EF1α promoter and can be detected in blood-, mosquito-, and liver stages. Importantly, luciferase signal in Py-GFP-luc infected mice correlated to liver stage burden as determined by qPCR, and increased over time as the liver stages progressed through development. This technique is very sensitive as liver stage infection could be observed at 44 hpi using inoculums of as few as 100 infectious salivary gland Py-GFP-luc sporozoites and as early as 16 hpi with a dose of 10^5^ infectious sporozoites. While this technique has been previously used in the *P. berghei* and *P. yoelii* YM (lethal strain) rodent models [4]–[6], this is the first time it has been described in the commonly utilized non-lethal *P. yoelii* XNL strain. The bioluminescent signal from the luciferase expressing *P. yoelii* YM parasites was not detectable at early time points (<42 hpi) in liver stage development [4], whereas in our study a bioluminescent liver stage signal could be readily detected at 16 hpi with 10^5^ Py-GFP-luc sporozoites. Furthermore, the sensitivity of the Py-GFP-luc bioluminescence system was at least 10 times greater when compared to luciferase expressing *P. yoelii* YM parasites, as the lowest detectable dose of these parasites was 1,000 sporozoites as compared to 100 Py-GFP-luc sporozoites. Differences in the strength of luciferase expression between these two *P. yoelii* strains may be due to insertion strategies of the luciferase constructs. While both constructs express luciferase under the EF1α promoter, the *P. yoelii* YM transgenic line was generated via a single cross-over recombination event in the ribosomal RNA small subunit gene locus [15], whereas Py-GFP-luc was generated via a double recombination event, replacing the dispensable S1 locus [12]. The luciferase cassette may be more stable in Py-GFP-luc than in the *P. yoelii* YM transgenic strain as reversion and excision of recombinant DNA is less likely following double recombination than a single recombination event.

Previous studies of *Plasmodium* liver stage burden in rodent models showed that different mouse strains exhibit differential susceptibility to different *Plasmodium* species [16]. *P. berghei* was shown to have a higher level of infection in C57BL/6 mice when compared to BALB/c mice [17], and *P. yoelii* more readily infects BALB/c when compared to C57BL/6 mice [18]. These early findings have led to the practice of pairing C57BL/6 mice with the *P. berghei* model and BALB/c mice with the *P. yoelii* model. These host-parasite combinations have discouraged use of the *P. yoelii* rodent model in functional immunology as most immune factor gene-deficient mice are available only on the C57BL/6 mouse background. However, using bioluminescent imaging and qPCR, we show that Py-GFP-luc has a similar infection profile in C57BL/6 and BALB/cJ mice. Furthermore, both strains require similar infectious doses, administered intravenously or by mosquito bite, to establish infection and blood stage patency in 100% of mice. These findings are comparable to those observed by Belmonte *et al*. who showed that infective doses of *P. yoelii* were similar between C57BL/6 and BALB/cJ mice when sporozoites were transmitted by i.v. injection [19]. More precise and detailed comparisons between *Plasmodium* species and host strain susceptibilities could be obtained by comparing bioluminescent signals from Py-GFP-luc and the luciferase expressing *P. berghei* parasites [6], [7].

Current immunization strategies to prevent malaria are focused on using either whole parasite, or subunit vaccination (reviewed in [3]). *In vivo* imaging of bioluminescent *Plasmodium* parasites has previously been used to evaluate immunity against malaria following immunization with blood stage parasites [4], as well as radiation attenuated sporozoites and infectious sporozoites with Chloroquine immunization protocols [5]. Here we show that the same method can be used to monitor immunity to GAP vaccination. Refined *in* *vivo* imaging protocols with Py-GFP-luc can now be established to determine at what time point parasites in the liver are killed following vaccination. This experiment can likely only be carried out using Py-GFP-luc parasites as the bioluminescent signal from both the *P. berghei*- and *P. yoelii*-YM luciferase expressing parasites cannot be detected until 24 hpi [4], [6].

Bioluminescent *in vivo* imaging of Py-GFP-luc parasites allows for quick and efficient quantitative monitoring of parasite growth in the liver in a non-invasive manner. This technique has clear benefits over the traditional invasive methods of parasite detection in the liver, such as qPCR and microscopy, as infection can be monitored in individual mice continuously throughout liver stage development without sacrificing the animals. This also allows the measurement of efficacy against additional challenges over time and thus will help determine duration of protection. Use of Py-GFP-luc parasites and bioluminescent imaging may greatly accelerate and streamline the process of identifying and analyzing future vaccine candidates but also drug candidates against the pre-erythrocytic stages of malaria parasites.

## Methods

### Ethics Statement

All murine studies have been performed according to the regulations of the Institutional Animal Care and Use Committee IACUC. Approval was obtained from the Seattle BioMed Experimental Animal Ethical Committee (OLAW assurance #A3640-01). Mosquito bite infections and bioluminescence imaging were performed on mice anesthetized either by intraperitoneal injection of Ketamine/Xylazine or by controlled inhalation of isofluorane. Following completion of experiments, mice were euthanized via administration of CO_2_ followed by cervical dislocation. Animals were monitored daily and every effort was made to minimize suffering.

### Mice

Female Swiss Webster mice were purchased from Harlan Laboratories and female BALB/cJ and C57BL6/J mice, six to eight weeks of age, were purchased from Jackson Labs.

### Parasite Growth and Sporozoite Isolation

Female six-to-eight week old Swiss Webster (SW) mice were injected with blood stage Py-GFP-luc or *P. yoelii* 17XNL wild type (WT) parasites to begin the growth cycle. The infected mice were used to feed female *Anopheles stephensi* mosquitoes after gametocyte exflagellation was observed. Ten days after the blood meal, 15–20 mosquitoes were dissected to evaluate midgut oocyst formation. At day 14 or 15 post-blood meal, salivary gland sporozoites were isolated and harvested as previously described [10].

### Generation of Py-GFP-luc Parasites

*P. yoelii* 17XNL genomic DNA (gDNA) was used as a template to amplify a 0.5 kb fragment of the 3′UTR and a 0.5 kb fragment of the 5′UTR of *PyS1* using oligonucleotide primers PyS1-1 EcoRV forward (F) and PyS1–2 ApaI reverse (R) and PyS1–3 ApaI F and PyS1–4 NotI R (primer sequences provided in Table S2). PyS1–2 R and PyS1–3 F contained a unique ApaI site and overlapping sequences. The 5′UTR and 3′UTR fragments were then combined by splicing by overlapping extension (SOE) PCR, and the resulting fragment was inserted into the plasmid b3D.DT.H Db-DsRed [20] between EcoRV and NotI restriction enzymes sites. The plasmid was then digested with KpnI and AflII to replace the red fluorescent protein cassette with the green fluorescent protein-luciferase cassette derived from pL1063. This plasmid was obtained through the MR4 as part of the BEI Resources Repository, NIAID, NIH: *Plasmodium berghei* pL1063, MRA-852, deposited by AP Waters. The resulting plasmid was digested with ApaI between the 5′ and 3′ UTRs to linearize the plasmid for transfection. Transfection of *P. yoelii* 17XNL parasites using the Amaxa Nucleofector device (Amaxa GmbH, Germany), resistant parasites selection and recombinant parasite cloning by serial limiting dilutions were all conducted as described elsewhere [21]. To confirm the targeted deletion and the genetic recombination, integration-specific gDNA PCR amplification of the *PyS1* locus was generated using the specific primers combinations 5′test (PyS1TestF and T7R) and 3′test (PyS1TestR and TgF). The *S1* open reading frame (ORF) was amplified using the primers S1ORFtestF and S1ORFtestR (primer sequences provided in Table S2).

### Infection Assays

BALB/cJ and C57BL/6 mice were infected with Py-GFP-luc or wild type *P. yoelii* salivary gland sporozoites either by intravenous tail-vein injection (i.v.) or by infectious mosquito bite. For i.v. injections, salivary gland sporozoites were enumerated and suspended in RPMI prior to injection of 4–100,000 sporozoites per mouse as described in the figure legends. For mosquito bite infection, animals were anesthetized with Ketamine/Xylazine and were placed on a feeding cage containing 5, 15, or 40 infected mosquitoes. Mosquitoes were allowed to feed on the mouse for 10 minutes total. Mice were lifted every minute and rotated amongst feeding cages every two minutes to ensure that all the mice were equally exposed to infected mosquitoes. Following infections, a subset of mosquitoes were dissected and sporozoites were enumerated to determine the level of mosquito infection.

### Real Time *in vivo* Imaging of Liver Stage Development in Whole Bodies of Live Mice

Luciferase activity in animals was visualized through imaging of whole bodies using the IVIS Lumina II animal imager (Caliper Life Sciences, USA) as previously described [4], [6], [22]. The bellies of C57BL/6 mice were shaved prior to imaging in order to minimize the absorption of light by the highly pigmented fur (data not shown). BALB/cJ mice were not shaved except when liver burdens were being compared directly to infected C57BL/6 mice (Figure 5). Mice were injected with 100 µl of RediJect D-Luciferin (Perkin Elmer) intraperitoneally prior to being anesthetized using the isofluorane-anesthesia system (XGI-8, Caliper Life Sciences, USA). Animals were kept anesthetized during the measurements, which were performed within 5 to 10 minutes after the injection of D-luciferin. Bioluminescence imaging was acquired with a 10 cm FOV, medium binning factor and an exposure time of 1 to 5 minutes. Quantitative analysis of bioluminescence was performed by measuring the luminescence signal intensity using the ROI settings of the Living Image® 3.0 software. ROIs were placed around the abdominal area at the location of the liver. ROI measurements are expressed as total flux (p/s).

### Immunization Assays

BALB/cJ mice were infected i.v. with either PBS or 5×10^4^ *Py-fabb/f-* salivary gland sporozoites. One group of mice was immunized with two doses of *Py-fabb/f-* three weeks apart and one group was immunized with one dose of *Py-fabb/f-*. All mice were challenged four weeks after the final immunization with 5×10^4^ Py-GFP-luc sporozoites. Bioluminescence was measured as described above.

### Luminescence of Py-GFP-luc Sporozoites, Oocysts and Infected RBCs

#### Infected RBC

Parasitemia in SW mice infected with Py-GFP-luc was determined by Giemsa-stained thin blood smear. Approximately 100 µl of infected blood was drawn and was serially diluted at a ratio of 1∶4 in RPMI. Thirty-five µl of diluted blood was plated in triplicate on a white 96 well luminometer plate (VWR) and mixed with an equal volume of Bright-Glo luciferase assay substrate (Promega). Luminescence was measured over a 10 second time period with a CentroXS^3^ LB 960 luminometer.

#### Sporozoites

Py-GFP-luc sporozoites were dissected from mosquito salivary glands and diluted to a concentration of 1,000 sporozoites/µl in RPMI. Sporozoites were serially diluted 1∶10 in RPMI. Bioluminescence of 50,000, 5,000 and 500 sporozoites in 50 µl of suspension was measured.

#### Midgut oocysts

To measure luciferase activity of Py-GFP-luc oocysts, midguts were dissected from infected mosquitoes and were categorized by the approximate number of oocysts per midgut. Categories consisted of uninfected, between 1 and 10 oocysts/midgut (1<10), between 11 and 100 oocysts/midgut (11<100), and greater than 100 oocysts/midgut (>100). Each midgut was put into a well on a luminometer plate with 35 µl of RPMI and luciferase activity was measured as described above. The same plate was then visualized with the IVIS with 10 cm FOV, small binning factor and an exposure time of 10 seconds.

### Quantitative PCR (qPCR)

Total RNA of Py-GFP-luc infected livers was extracted using TRIzol reagent (Invitrogen) and treated with TURBO DNase (Ambion). CDNA synthesis was performed using the Super Script III Platinum two-step qPCR kit according to the manufacturer’s instructions (Invitrogen). Primers used for amplification of 18S rRNA and GAPDH from cDNA are documented in Table S1. All PCR amplification cycles were performed at 95°C for 30 s for DNA denaturation, and 60°C for 4 min for primer annealing and DNA strands extension. For semi-quantitative PCR (qPCR), a standard curve was generated using 1∶4 dilutions of a reference cDNA sample for PCR amplification of all target PCR products. Experimental samples were compared to this standard curve to give a relative abundance of transcript. All signals were normalized to average abundance of transcript from a reference sample as designated in the figure legends.

### Statistics

Kaplan-Meyer patency curves between BALB/cJ and C57BL/6 mice were compared via log rank Mantel-Cox test. Correlation coefficients between qPCR and Luminescence measures of liver stage burden were determined using the Pearson’s r test. All other comparisons were two-tailed Student’s T test. Statistical significances are as follows: *0.05≥p>0.01; **0.01≥p>0.001; ***0.001≥p>0.0001; non-significant (ns) p≥0.05.

## Supporting Information

Figure S1 **Generation of the luciferase expressing** ***P. yoelii*** **XNL parasite.** A cassette coding for a GFP-luciferase fusion protein (GFP-luc) under control of the EF1α promoter was inserted into the *P. yoelii* (Py) genome using double homologous recombination of the 5′ and 3′ UTR regions at the Py*-S1* locus. The open reading frame of S1, which is dispensable, was replaced with a selectable drug resistance marker (*DHFR/TS*: the *DHFR/TS* gene from *Toxoplasma gondii* - a positive selection marker; AMP: Ampicillin resistance gene for bacterial selection) in addition to the GFP-luc cassette. (B) The mixed population of wild type and transgenic parasites (TRP1) was cloned by serial dilution in SW mice. Genotyping gel shows successful integration on the 3′ (3′ test) and 5′ (5′ test) flanks, as well as the loss of the *Py-S1* open reading frame in four independent clones. The mixed population TRP1 shows the presence of the *Py-S1* open reading frame (ORF) and integration events, whereas only the *Py-S1* ORF can be detected in DNA from wild type *P. yoelii.*
(TIF)

Figure S2 **Py-GFP-luc parasites develop normally during blood stages.** Blood stage growth curve. One million control (Py) or Py-GFP-luc blood stage parasites were injected into BALB/cJ mice and parasitemia in the blood was measured daily until all parasites were cleared.
(TIF)

Table S1 **Comparisons of wild type** ***P. yoeli*** **and Py-GFP-luc pre-erythrocytic stages.**
(TIF)

Table S2 **Description of primers utilized.**
(TIF)
