# Supplementary figures and images for: Quantitative Bioluminescent Imaging of Pre-Erythrocytic Malaria Parasite Infection Using Luciferase-Expressing Plasmodium yoelii
Source: PLoS One. 2013 Apr 11;8(4):e60820. doi: 10.1371/journal.pone.0060820 (PMC3623966; doi:10.1371/journal.pone.0060820)

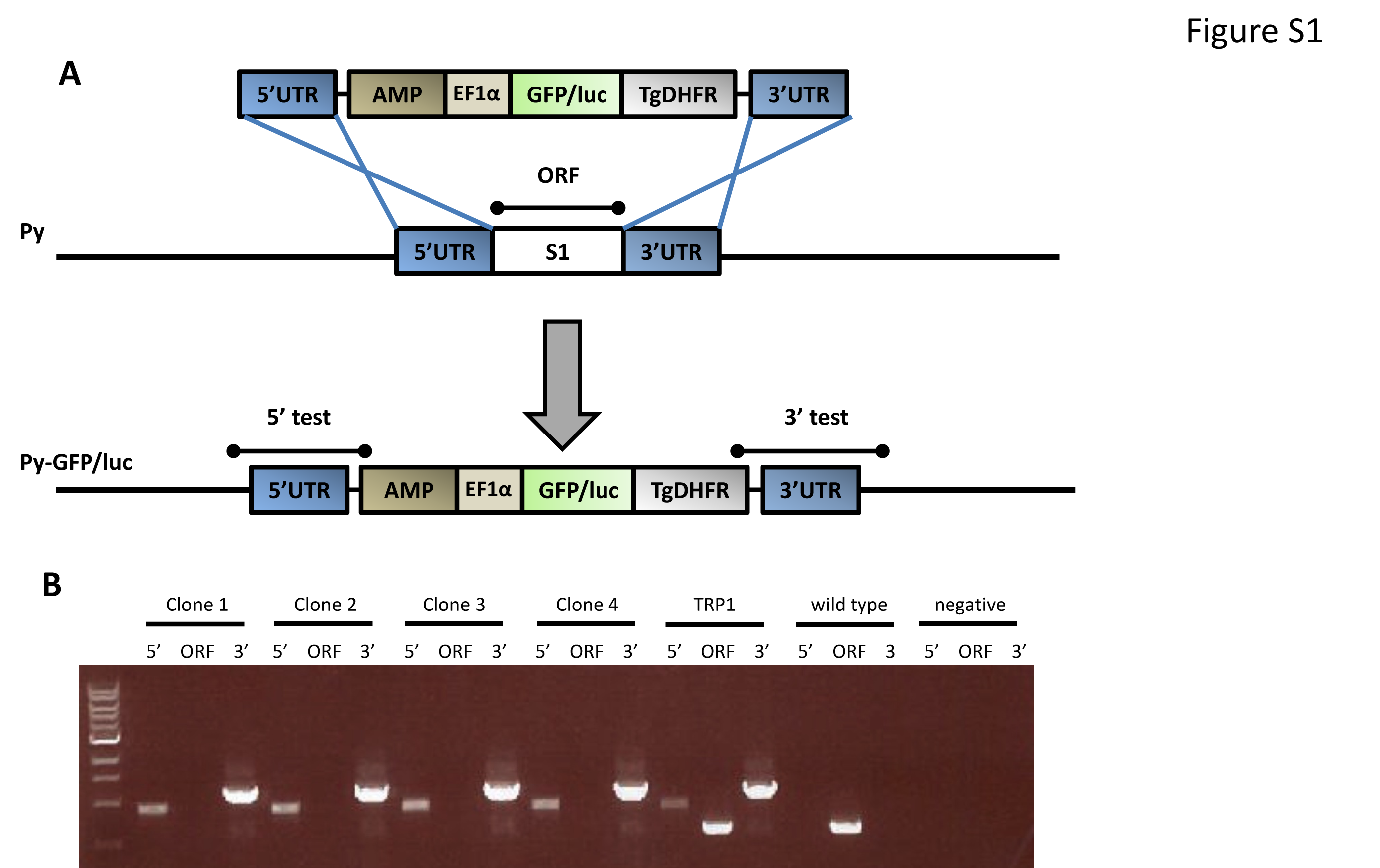

Supplement: Figure S1 — Generation of the luciferase expressing P. yoelii XNL parasite. A cassette coding for a GFP-luciferase fusion protein (GFP-luc) under control of the EF1α promoter was inserted into the P. yoelii (Py) genome using double homologous recombination of the 5′ and 3′ UTR regions at the Py-S1 locus. The open reading frame of S1, which is dispensable, was replaced with a selectable drug resistance marker (DHFR/TS: the DHFR/TS gene from Toxoplasma gondii - a positive selection marker; AMP: Ampicillin resistance gene for bacterial selection) in addition to the GFP-luc cassette. (B) The mixed population of wild type and transgenic parasites (TRP1) was cloned by serial dilution in SW mice. Genotyping gel shows successful integration on the 3′ (3′ test) and 5′ (5′ test) flanks, as well as the loss of the Py-S1 open reading frame in four independent clones. The mixed population TRP1 shows the presence of the Py-S1 open reading frame (ORF) and integration events, whereas only the Py-S1 ORF can be detected in DNA from wild type P. yoelii. (TIF) [file pone.0060820.s001.tif]

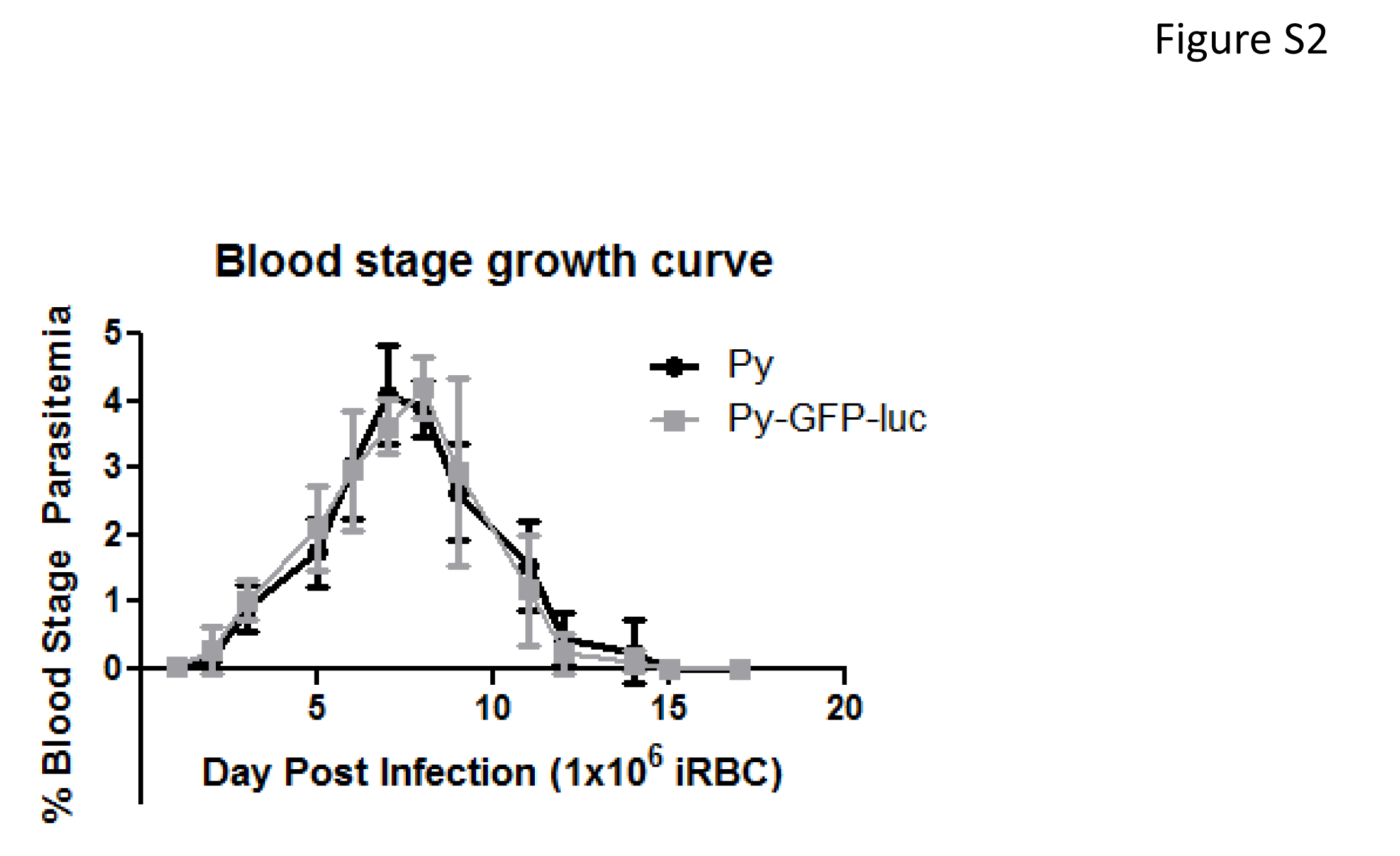

Supplement: Figure S2 — Py-GFP-luc parasites develop normally during blood stages. Blood stage growth curve. One million control (Py) or Py-GFP-luc blood stage parasites were injected into BALB/cJ mice and parasitemia in the blood was measured daily until all parasites were cleared. (TIF) [file pone.0060820.s002.tif]

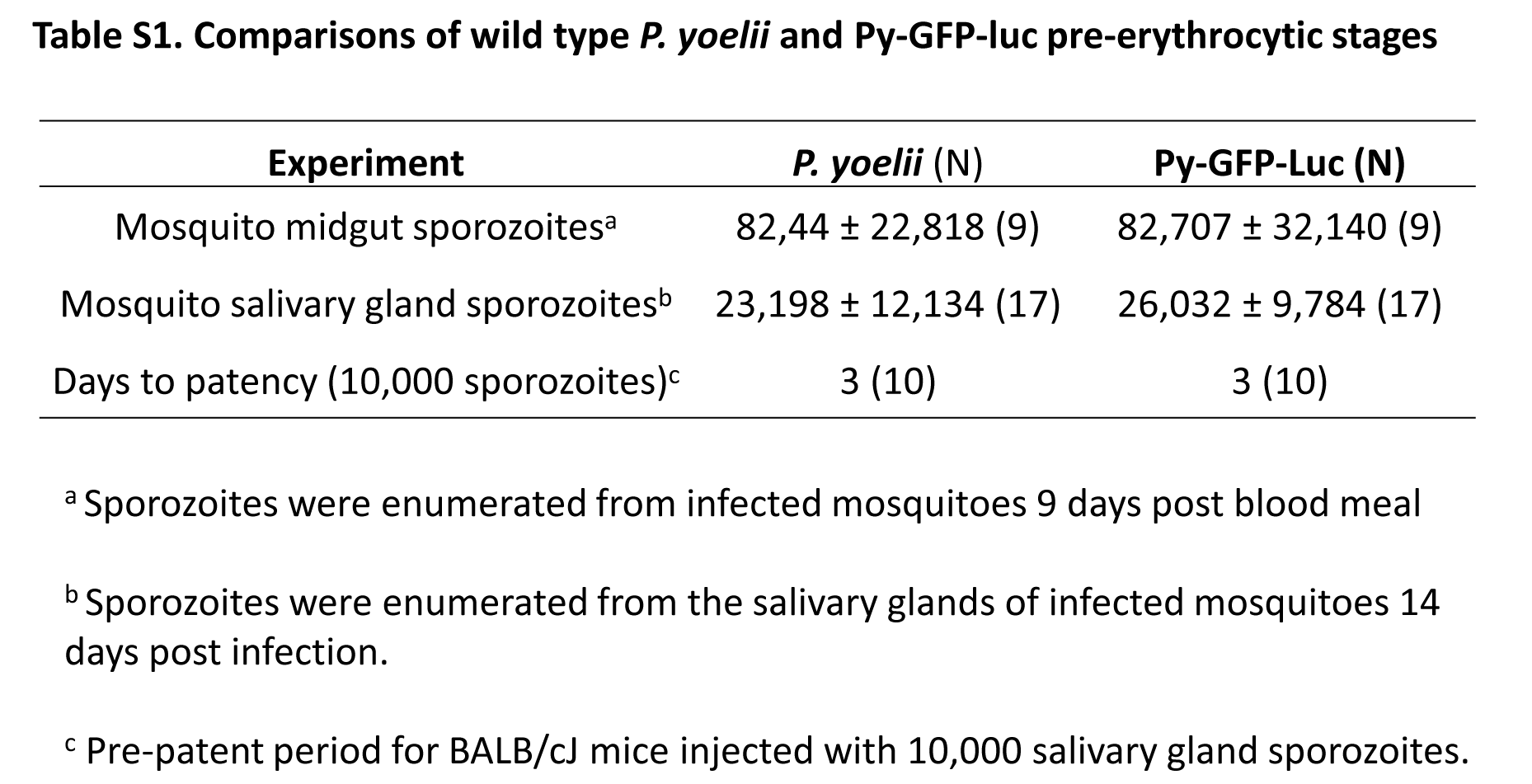

Supplement: Table S1 — Comparisons of wild type P. yoeli and Py-GFP-luc pre-erythrocytic stages. (TIF) [file pone.0060820.s003.tif]

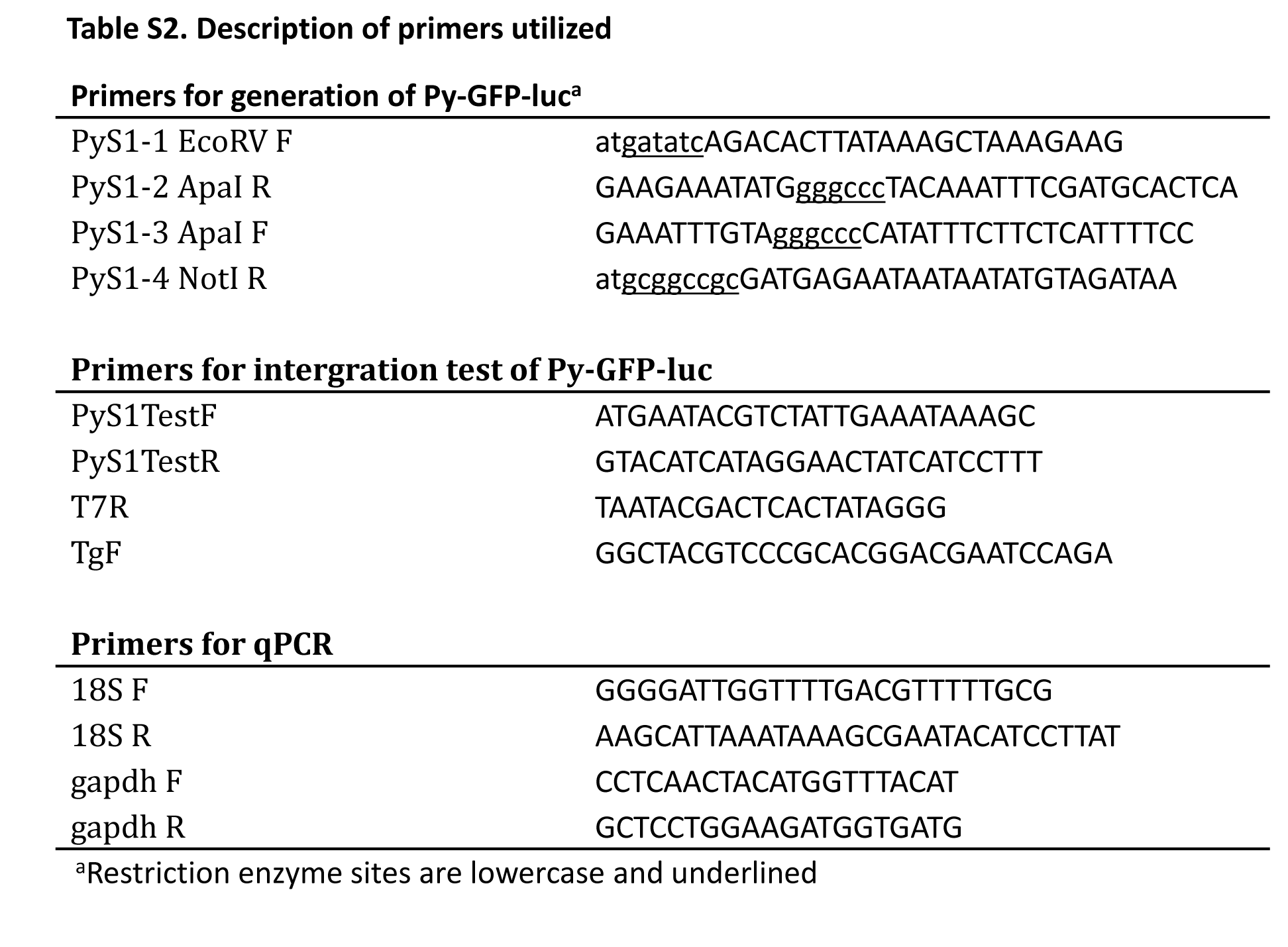

Supplement: Table S2 — Description of primers utilized. (TIF) [file pone.0060820.s004.tif]
